# The impact of emotion regulation on the establishment of the therapeutic alliance in post-divorce group intervention: A multilevel approach

**DOI:** 10.1371/journal.pone.0312293

**Published:** 2024-10-18

**Authors:** Irati Alvarez, Marta Herrero, Ana Martínez-Pampliega

**Affiliations:** 1 Department of Psychopedagogy, Begoñako Andra Mari Teacher Training University College, BAM, Derio, Biscay, Spain; 2 Faculty of Health Science, University of Deusto, Bilbao, Biscay, Spain; Government Medical College and Hospital, INDIA

## Abstract

**Objective:**

The present study focuses on the impact of emotion regulation on the establishment of the therapeutic alliance in a context of post-divorce group intervention.

**Method:**

The study involved 177 divorced parents and 60 therapists and was developed through multilevel path analysis.

**Results:**

The data showed an association between emotion regulation and therapeutic alliance across the difficulties of adaptation to divorce, although the results were different from the perspective of the participant and of the therapist and between the individual and the group level.

**Conclusion:**

The study highlights the relevant role of emotion regulation in post-divorce adaptation and in the success of the establishment of the therapeutic alliance, while clarifying the formation of the alliance from the viewpoints of the individual and the therapist. The study also highlights the need to understand the alliance at both the individual and the group level, in order to design therapeutic interventions.

## Introduction

In recent decades, divorce cases have increased internationally. In Europe, it is estimated that around 1 million families go through a situation of divorce, while in Spain, a divorce rate of 58% is estimated, and specifically, Spain is one of the European countries with the highest divorce rate [1]. In addition, 60% of parents who decide to divorce have children in their care [2]. Along with these statistics, there is an extensive bibliography that includes the impact of divorce on the mental health of parents and children [3–5]. All of this has promoted the development of intervention programs aimed at facilitating parents’ adaptation to divorce in order to decrease their symptoms and reduce the psychological impact of divorce on all members of the family [6,7].

Multiple intervention programs have been designed with this aim, some of which have efficacy studies [8,9]. In Spain, only the Egokitzen program has efficacy studies [10,11]. In order to understand the impact of these therapeutic interventions, research has sought to identify the relevant variables, among which the therapeutic alliance has been highlighted [12].

The most widely employed conceptualization of therapeutic alliance [13] that has the most influence in psychotherapy [14] is the trans-theoretical proposal of [15], which is characterized by establishing a collaborative attitude between patient and therapist in the agreement on the tasks and objectives of the therapy. It is widely recognized the influence of the therapeutic alliance in intervention outcomes [16–18].

After the initial decades of research on the relationship between the therapeutic alliance and the outcome of interventions, the current focus is on exploring the establishment of the therapeutic alliance in greater depth.

In this sense, there is emerging evidence of associations between therapeutic alliance and emotion regulation [19,20]. The emotion regulation refers to the process through which individuals modulate their emotions and modify their behavior to achieve goals, adapt to the context, or promote their well-being [21]. Higher levels of difficulties in emotion regulation are associated with lower therapeutic alliance [19,20,22,23]. Thus, the ability to regulate emotions might influence the establishment of therapeutic alliance [24]. Concretely, a person who is overwhelmed by their emotional experience may not be prepared to establish a relationship with the therapist [20].

Understanding the relation between these variables is especially relevant in the context of divorce, which has often been described as a process of stress [25,26] and seems to be linked to emotional dysregulation [26–28]. That is, those people with greater emotion regulation abilities seem to cope better with the stress of divorce, suffer lower levels of symptomatology [29,30], and have better post-divorce relations [28].

However, despite that the consideration of divorce as a very emotionally intense process is clearly accepted, research focused on understanding this emotional experience is scarce and there is even less research on its connection with the comprehension of the therapeutic alliance. Thus, the need to continue to deepen the study of emotion regulation in relation to therapeutic alliance, the process of adaptation to divorce and symptomatology in post-divorce group interventions seems obvious.

In the literature review, it is noteworthy that most of the research on the therapeutic alliance has focused on individual therapies [31–33] (and, although less frequent, also on family and couple therapies [34–36]. However, there are practically no studies analyzing the establishment of the therapeutic alliance in group settings [37] and none concerning divorce.

### The present study

The literature review has highlighted the relevance of some variables such as the therapeutic alliance in post-divorce intervention programs. As divorce is a stressful process, emotional regulation could play a key role in establishing the alliance. In addition, our comprehension of the alliance in a group context needs to be examined in further depth.

**Fig 1:**
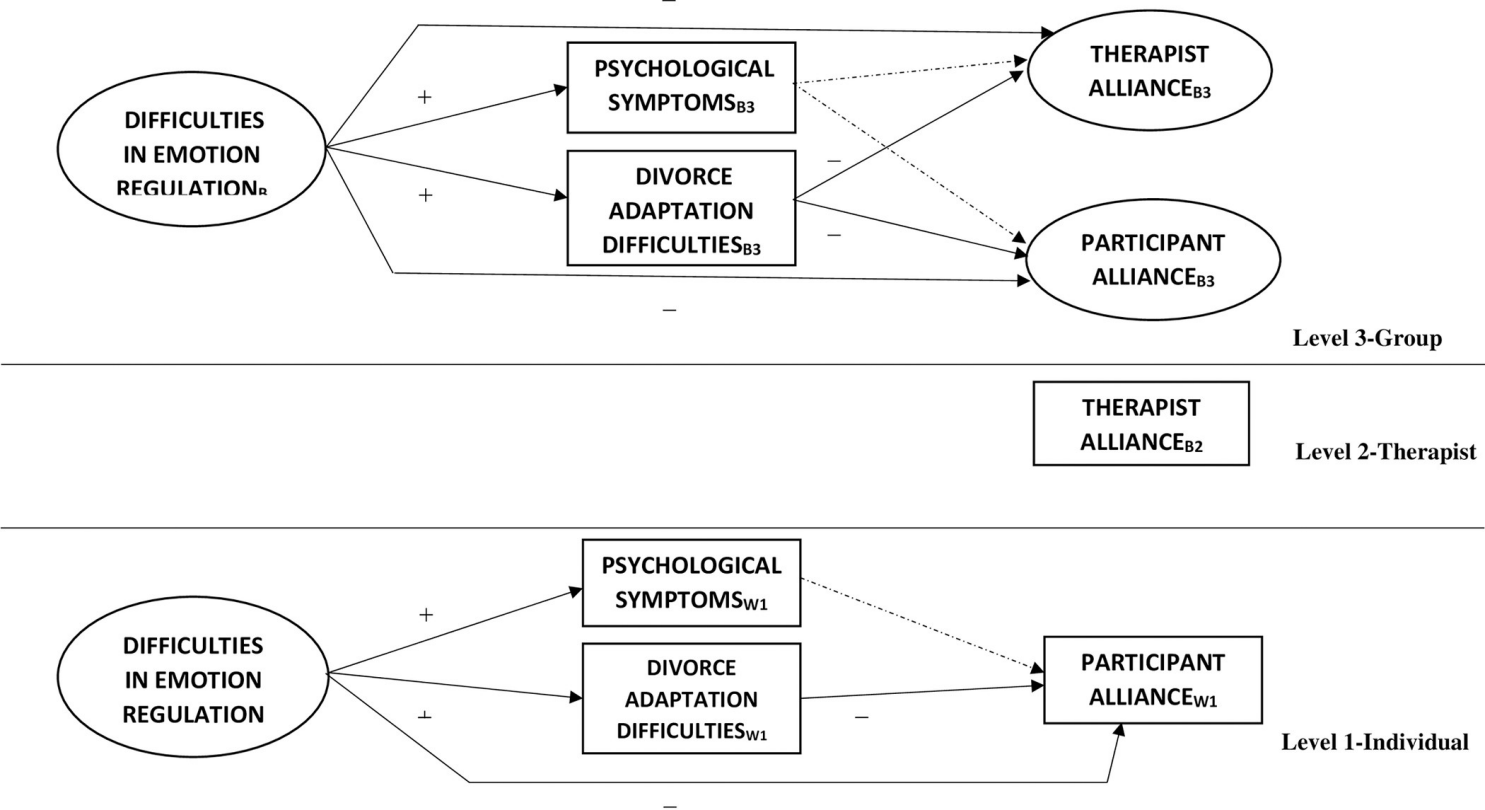
Hypothesized model. B3 = variable modeled at Level 3, B2 = variable modeled at Level 2, W1 = variable modeled at Level 1.

Thus, the objective of this study is to understand the impact of emotion regulation on the establishment of the therapeutic alliance in a context of post-divorce group intervention. In accordance with the results of the literature, the hypothesized model is presented in Fig 1.

This proposal has been operationalized through 1) the "Sistema de Observación de la Alianza Terapéutica en Intervención Familiar" or System for Observing Family Therapy Alliances (SOATIF or SOFTA; [38,39]) due to its wide recognition and the possibility to apply it in individual, family, couple, and group therapies [40] 2) multilevel analysis because it is appropriate to handle the nested structure of the data [41] 3) with parents participating in the post-divorce intervention program Egokitzen, which, as mentioned, has studies of efficacy and efficiency in Spain [10,11,42].

## Method

### Participants

Participation in the investigation was voluntary, and participants were ensured about the anonymity of the responses to the questionnaires. Participants were also informed of the possibility of dropping out of the investigation if they wished to do so. The investigation was approved by the ethics commission of the authors’ university; University of Deusto (ETK-7/16-17) and participants gave written informed consent.

The participants in the study are 177 parents involved in 30 intervention groups for separated or divorced parents, who attended 12 visitation centers (specialized resource to support families after their break-up), and their therapists, a total of 60. In order to clarify the terms, the parents attending the groups are hereinafter called “participants” and their therapists are named “therapists”.

With regard to the participants, the inclusion criteria for parents were: a) motivation to participate in a post-divorce intervention; b) to be divorced or separated c) not live with their ex-partner; d) not have a psychological disorder diagnosed; e) completing the questionnaires at two moments (pre-intervention questionnaires and alliance at the 3rd session of the intervention).

Participants’ average age was 40.32 years (*SD* = 7.14) and there were more women than men (63 vs. 37%, respectively). These parents had been married for an average of 10.44 years (*SD* = 6.51). Concerning time since divorce, 48% of the parents reported having separated/divorced more than three years ago, 24% between one and two years ago, 11% from two to three years ago, and the remaining 17% less than one year ago. The relations with the ex-partner were mainly non-existent or limited (40 vs. 26%, respectively), in 16%, they were very scarce, and in 9%, they were variable. Only 8% considered their relationship to be fluid.

The parents’ average number of children was 1.59 (SD = 0.85), and the presence of a single child (57%) or of two children (33%) was very frequent. The children’s mean age was 8.24 years (SD = 4.35). In 71% of the cases, the participating parent was the custodial parent, with 67% of the cases presenting exclusive maternal custody, 21% shared custody, and only in 9% exclusive paternal custody.

With regard to the therapists, their mean age was 40.05 years (*SD* = 8.96) and they were mostly women (86% female vs. 14% male). According to the inclusion criteria, they all had experience in addressing post-divorce problems and had participated in the specific training of the program.

### Pre-intervention measures

#### Adaptation to divorce

Adaptation to divorce was measured by the "Cuestionario de Adaptación al Divorcio-Separación" (CAD-S; [43]). This instrument evaluates the adaptation to divorce-separation and consists of four dimensions. In the present study, only the dimension of Psychological and Emotional Difficulties in the adaptation to divorce-separation was administered. It refers to feelings of anger towards the ex-partner, thoughts about never being able to overcome the breakup of the relationship, or never really believing in the separation, also describing obsessive interest in the ex-partner. The dimension is made up of 6 items rated on a Likert-type response format. In the present study, Cronbach’s alpha for the mentioned dimension was α = .74, and McDonald’s omega was ω = .77, indicating acceptable reliability.

#### Parental psychological symptomatology

Psychological symptomatology was measured by the *Symptom Checklist-90* (SCL-90; [44]), adapted and validated in Spanish by [45]. This questionnaire consists of nine dimensions evaluating clinical symptoms in adults. Four dimensions were used in this study: Interpersonal sensitivity, Depression, Anxiety, and Somatization. These four dimensions are rated on 44 Likert-type items. In this study, the alpha for these four subscales taken together was α = .96 and the omega was ω = .97, indicating very high internal consistency.

### Emotion regulation

Emotion regulation was measured through the *Difficulties in Emotion Regulation Scale (*DERS; [46], in the Spanish adaptation by [47]. This scale examines the difficulties that may emerge in the process of emotion regulation and consists of 25 Likert-type items, grouped in five dimensions: Non-acceptance, Lack of objectives, Impulsiveness, Lack of strategies, and Lack of clarity. It also has a global score for Emotion regulation. In this study, the reliability of the global scale was very high (α = .95, ω = .95).

### Intervention measures

#### Therapeutic alliance

Therapeutic alliance was measured through the *System for Observing Family Therapy Alliances* (SOFTA: [38,39]). This consists of 16 Likert-type items grouped in four dimensions: Engagement in the therapeutic process, Emotional connection with the therapist, Safety within the therapeutic system, and Shared sense of purpose within the family. In addition, the questionnaire provides of a global score of Therapeutic alliance. The questionnaire is presented in two versions: one to be answered by the patients and the other to be completed by the therapists. In the patient version, the respondent must respond to the content of each item referring to their alliance with the therapist, and in the version for the therapists, the items are completed according to their perception of alliance with the group. In this study, for patients’ therapeutic alliance, the internal consistency of the scale was high (α = .84, ω = .86) and for therapists’ therapeutic alliance, the mean reliability among therapists was acceptable (α = .66, ω = .71).

### Procedure of the present study

**Fig 2:**
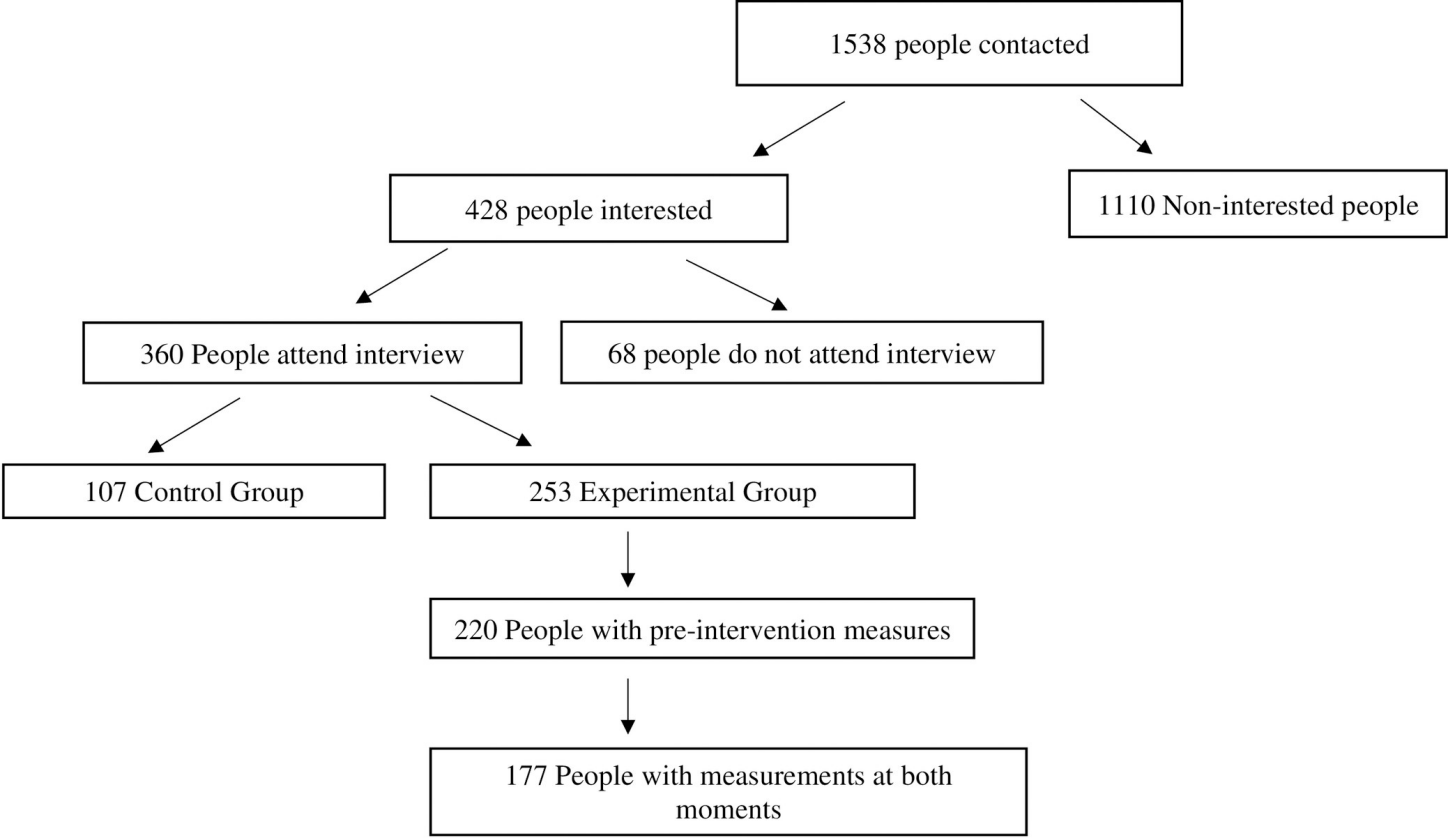
Flow chart of study sample.

The present study was performed at 12 nationwide visitation centers. We contacted 1538 people to ask them about their interest in participating in the intervention program, of whom 428 reported being interested and were summoned to a personal interview. This personal interview was attended by 360 people, who were informed about the program and who signed the informed consent. However, 107 could not meet the conditions of participation and be included in the experimental group (due to working hours, shift work, family conciliation, etc.). Two hundred twenty people completed the questionnaires on the pre-intervention variables of this study, but 20% (n = 43) did not complete the second step in the 3rd session, either due to not attending this particular session or to dropping out of the intervention group because of their job. So finally, 177 parents completed pre-intervention and intervention measures of the study. A flow chart of the parents participating in the study is presented in Fig 2.

The professionals in these visitation centers were trained by the members of the research team to evaluate and implement the intervention program. We applied the Egokitzen program [10,42], which is a program focuses on the relevant factors of interparental conflict in divorce situations, especially those that minimize the impact of divorce and promote resilience. It consists of 11 sessions of 1.5 h, which address three thematic areas from a systemic approach to family functioning: divorce and its impact, interparental conflict, and parenting. The sessions are designed to actively engage participants through role-playing, debates, and group activities. Each intervention group was led by two trained professionals, at least one of whom was a psychologist, and the average number of participants in the groups was 5.9.

Regarding data collection, participants and therapists both completed the questionnaires (from January 2017 to December 2019). With regard to the participants, before starting the intervention group, they completed three instruments individually (DERS, SCL-90, and CAD-S). After the third session, (session identified as relevant for the establishment of the therapeutic alliance; [48,49]), the patient version of the SOFTA questionnaire was administered. The therapists only completed their version of the SOFTA questionnaire after the third session.

### Analysis procedure

As a preliminary analysis, we verified the absence of significant differences in sociodemographic and target predictive variables between the participating group and those not included for lack of the second measurement. Data loss was small, about 6% maximum in most indicators. In addition, Little’s MCAR test indicated that the missing value patterns were completely random, χ^2^(5531) = 5147.15, p > .999, so pairwise analysis was carried out.

After the preliminary analyses, the hypothesized model was tested (see Fig 1). For this purpose, we used multilevel path analysis in a structural equation framework with Mplus 7.0 [41] with Robust Maximum Likelihood. Specifically, we analyzed a model with three levels in which the individual data (Level 1) were nested within therapists (Level 2) and intervention groups (Level 3).

To perform the analysis, two models were compared: (1) the null model, and (2) the model with all the predictors (Model 1). The null model estimates the variance attributable to each level of analysis and establishes the base model for the comparison of subsequent models. From this model, we estimated the Intraclass Correlation Coefficient (ICC) of the alliance from the viewpoint of the participant and of the therapist to determine the degree of similarity between the data within each "cluster" or grouping level (i.e., therapists and/or group clusters) [50].

Model 1 included the relationships shown in Fig 1. Based on the variance observed in the null model, participant alliance was modeled as a dependent variable at Level 1-Individual and Level 3-Group, while the therapist alliance was modeled at Level 2-Therapist and Level 3-Group. The variable difficulties in emotion regulation was modeled as a latent factor with the five dimensions as indicators. To control for Level 1, the match between the participant’s and the therapist’s gender was included and, at Level 2, therapeutic experience.

Following the recommendations of [51], the variables measured at Levels 2 or 3 were grand-mean centered at the corresponding level and the variables modeled to Level 1 were group-mean centered. To test these indirect effects, we followed the recommendations of [52].

For the comparison between models, the following absolute indicators of good fit were considered: the ratio of chi-square (χ^2^) and degrees of freedom lower than 3, Comparative Fit Index (CFI) and Tucker-Lewis Index (TLI) greater than .90, and Root Mean Squared Error of Approximation (RMSEA) below .08. As relative fit indicators, lower values of Akaike Information Criterion (AIC) and Bayesian Information Criterion (BIC) were considered to be indicators of better fit [53]. The likelihood ratio test was used to determine whether the alternative model led to a significant increase in fit [54].

The data set of the study is available at http://dx.doi.org/10.17632/jmppy4h8k2.1

## Results

The descriptive statistics and correlations between the variables under study are included in Table 1.

**Table 1:** Descriptive statistics and correlations between study variables.

		*SD*			Correlations				
Study variable	*M*	Level 1	Level 2	Level 3	1	2	3	4	5
1. Divorce adaptation difficulties	1.84	0.78	--	0.40	(.74)	.51***	.54***	.11	
2. Psychological symptoms	40.12	29.11	--	14.39	.51**	(.96)	.62***	-.10	
3. Difficulties in emotion regulation	8.83	3.38	--	1.74	.48**	.76***	(.95)	-.13	
4. Patient alliance	4.29	0.33	--	0.22	< .01	.23	.16	(.84)	
5. Therapist alliance	4.24	--	0.20	0.28	-.21	.22	.25	.47**	(.66)

*Note*. Correlations above the diagonal are Level 1 correlations (i.e., Participants, n = 177), and Level 3 correlation are shown below the diagonal (i.e., Groups, n = 30). Correlations for Level 2 (i.e., therapist units, n = 60) are not provided because only therapist alliance was modeled at this level. Alpha coefficients are displayed in parentheses.

**p < .01.

***p < .001.

### Null model

This first model showed that the ICC of the therapeutic alliance reported by the therapists was .33 at Level 2, whereas, at Level 3, it was .67. This indicates that 67% of the variance of the therapist’s alliance was related to differences between groups, and 33% to differences between therapists.

This model showed that the ICC of the therapeutic alliance reported by participants at the level of therapists was < .01, at the group level, it was .30. and, at the individual level, it was .70. This information indicates that the participant’s alliance presented 70% variability among participants, and 30% among groups, while it presented a near-zero variability among therapists. Therefore, the participant’s alliance was modeled only at Levels 1 and 3.

Likewise, the psychological and emotional difficulties of adaptation to divorce had an ICC < .01 at the therapist level, and .07 and .14, respectively, at the group level. Therefore, the remainder of the variability was distributed between the individual and group levels. Specifically, psychological symptomatology showed a variability of 93% at the individual and of 7% in the groups, whereas the emotional and mental difficulties of adaptation to divorce showed a variability of 86% at the individual and of 14% in the groups. As in both variables, the variance at Level 2-therapists was close to zero, they were not modeled at that level but at Levels 1 and 3.

The likelihood ratio test indicated that the null model significantly increased the fit with respect to a non-multilevel model, χ^2^(89) = 5010.97, *p* < .001, or a model with only two levels, χ^2^(8) = 471.73, *p* < .001. The zero-model fit indicators were poor, χ^2^(83) = 923.69; χ^2^/*df* = 11.26; AIC = 15927.13; BIC = 16081.90; CFI < .01; TLI = < .01; RMSEA = .17.

These data indicate that the variance due to the hierarchical structure of the data was important and that, therefore, multilevel analysis is an adequate method of analysis. This model was established as the base model of comparison.

### Model 1: Direct effects on therapeutic alliance

**Fig 3:**
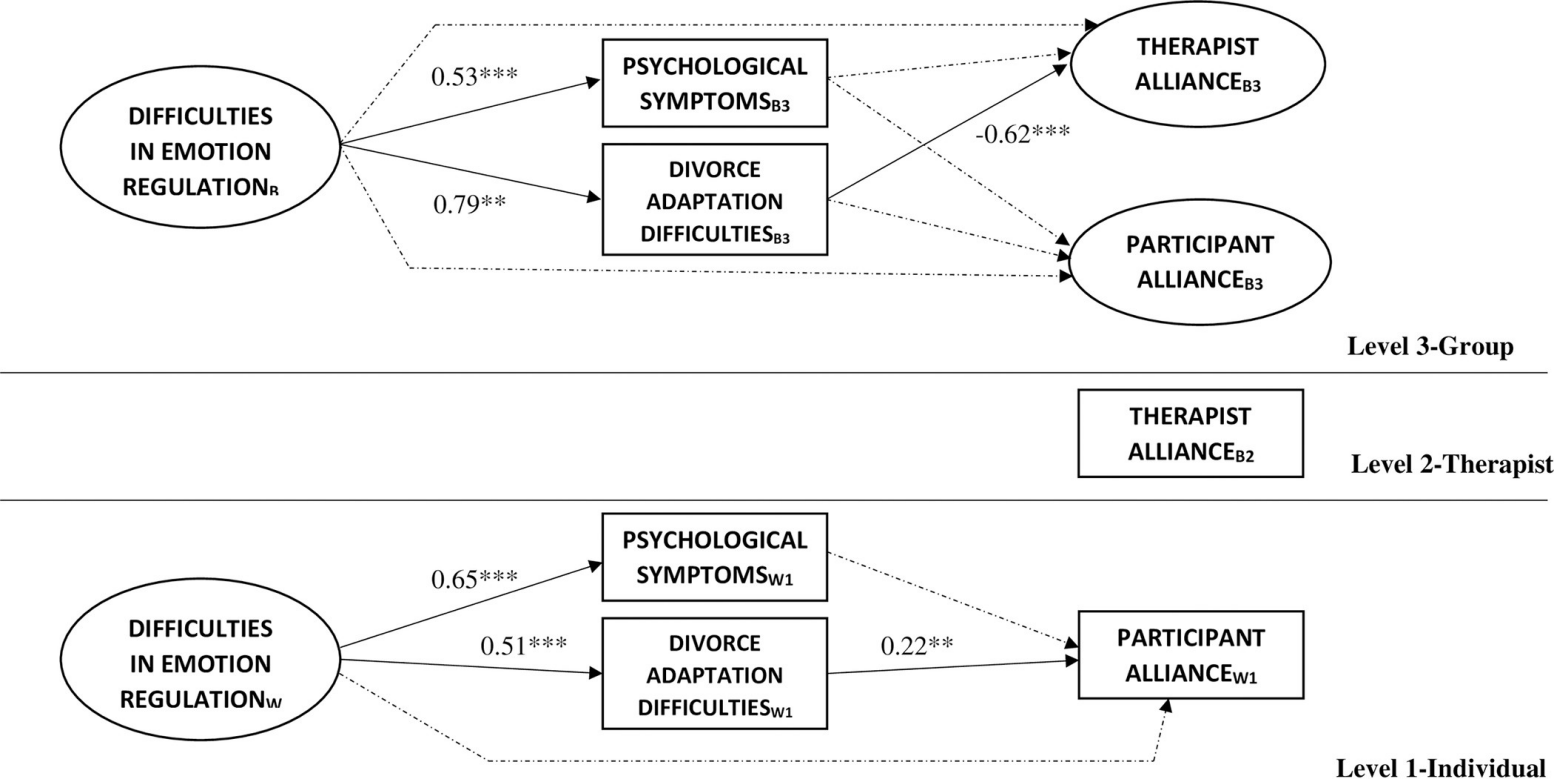
Multilevel standardized coefficients of significant Model 1 paths. Dashed lines represent non-significant paths. B3 = variable modeled at Level 3, B2 = variable modeled at Level 2, W1 = variable modeled at Level 1. ** *p* < .01, *** *p* < .001.

Model 1 (see Fig 3) significantly increased the fit with regard to the null model, χ^2^(30) = 2534.60, *p* < .001. In fact, all the fit indicators of this model were very good and better than those of the null model, χ^2^(52) = 93.18; χ^2^/*df* = 1.79; AIC = 13438.53; BIC = 13682.30; CFI = .95; TLI = .93; RMSEA = .05.

As shown in Table 2, the participants’ emotion regulation difficulties were related to higher levels of psychological symptomatology and emotional and mental difficulties of adaptation to divorce at Levels 1 and 3. That is, the people and groups of participants with more emotion regulation difficulties tended to present more symptomatology and more psychological difficulties of adaptation to divorce.

**Table 2:** Standardized multilevel model estimates of direct effects on participant and therapist’s Alliance.

		Dependent variable						
		Participant alliance				Therapist alliance		
Level	Independent variable	β	SE	95% CI		β	SE	95% CI
Level 1 –Participant	Same gender	0.08	0.15	[-0.14, 0.30]				
	Different gender	0.13	0.13	[-0.10, 0.25]				
	Divorce adaptation difficulties	0.22**	0.08	[0.03, 0.18]				
	Psychological symptoms	-0.11	0.39	[<-0.01, <0.01]				
	Difficulties in emotion regulation	-0.17	0.12	[-0.04, <0.01]				
Level 2 –Therapist	Therapeutic experience					0.20	0.16	[-0.02, 0.12]
Level 3 –Group	Divorce adaptation difficulties	-0.04	0.26	[-0.31, 0.26]		-0.62***	0.17	[-0.78, -0.12]
	Psychological symptoms	0.08	0.38	[-0.01, 0.01]		0.03	0.30	[-0.01, 0.01]
	Difficulties in emotion regulation	0.19	0.38	[-0.07, 0.12]		0.58	0.31	[-0.01, 0.19]

*Note*. Participant-therapist match on gender was modeled as two dummy variables (i.e., same gender and different gender).

**p < .01

***p < .001.

In the prediction of the alliance, it was observed that the psychological symptomatology had no significant effect on the alliance either of the participant or of the therapist, at any level. However, the psychological and emotional difficulties of adaptation to divorce did show a significant effect, although different in relation to the alliance perceived by the participant or by the therapist. With regard to the participants’ alliance, it was observed that its effect on alliance of the psychological and emotional difficulties of adaptation to divorce was significant at Level 1 but not at Level 3.

With regard to the therapist’s alliance, it was observed at Level 3 that the psychological and emotional difficulties of adaptation to divorce were related to lower therapeutic alliance, in the opposite direction as that observed in relation to the participants’ alliance. Likewise, as seen in Table 2, none of the control variables had a significant effect.

This model explained 6% of the variance of participant-perceived therapeutic alliance at Level 1 and 6% at Level 3. Likewise, model explained 35% of the variance of therapist-perceived therapeutic alliance at Level 3.

### Model 1: Indirect effects on the therapeutic alliance

Based on the observations in Model 1, the indirect effect of emotion regulation difficulties in participants’ and therapists’ therapeutic alliance was tested through the psychological and emotional difficulties of adaptation to divorce. The indirect effects were not tested through psychological symptomatology because it had no effect on the therapeutic alliance.

The results of these analyses showed that the indirect effects of emotion regulation on the participant’s therapeutic alliance (β = 0.01, SE < .01, *p* = .021, 95% CI (Confidence Interval) [<0.01, 0.02]) and on that of the therapist (β = -0.05, SE = 0.02, *p* = .003, 95% CI [-0.08, -0.02]) were both significant, although with the opposite sign.

## Discussion

The present study, whose main objective was to analyze the impact of emotion regulation on the establishment of the therapeutic alliance in a post-divorce group intervention, has provided relevant results in support of emotion regulation. The results have shown the importance of differentially understanding alliance in a group setting and the need to take into account both participants’ and therapists’ perceptions. However, the data have not fully confirmed the initial approach, insofar as the relationship between emotion regulation difficulties and the therapeutic alliance were only indirect. The results will be presented in detail.

Firstly, regarding the impact of emotion regulation, the data underline the importance of understanding this variable in relation with family conflicts, as well as in interventions, people’s well-being, and post-divorce interpersonal relationships. These relationships, mainly verified in other contexts [28,55,56], have been supported in the context of high interparental post-divorce conflict, thus contributing to understanding the adaptation to divorce and the impact of interventions on the therapeutic alliance. However, this relationship was not direct, but occurred instead through its association with the psychological and emotional difficulties related to adaptation to divorce. Hence, these explanatory mechanisms could be addressed to achieve greater therapeutic alliance. Therefore, it is relevant to study a variable of the divorce context, that is, the specific psychological and emotional difficulties undergone by parents who decide to divorce.

Secondly, the data showed that the relationship between emotion regulation and the therapeutic alliance occurred both from the perspective of the participant and of the therapist, although differentially. That is, parents and groups with more emotion regulation difficulties and also with greater psychological and emotional difficulties of adaptation to divorce achieve a greater therapeutic alliance with the therapist. However, in such situations, therapists perceive a lower therapeutic alliance at the group level. As the existing literature shows, the divorce process is a stressful situation for parents [25], and the need for help felt by the parents with the greatest difficulties when facing such stress could lead them establish a greater therapeutic alliance. On the contrary, for therapists, it may be overwhelming to stand before a group with serious difficulties both in emotion regulation and adaptation to divorce, which may lead them to perceive a lower therapeutic alliance with this kind of groups. The differences between the two perceptions found in our study are in accordance with the results of other studies [57,58]. Therefore, we emphasize the need to take into account both perspectives (participant and therapist), and not only those of individual modalities, couples, or family interventions, as has been the rule to date [59,60]. We should also consider the therapeutic alliance when planning the design and evaluation of interventions carried out in groups.

Thirdly, the results obtained also seem to show that there are differences between the processes observed at the individual and group level. Thus, the data are consistent with the approach of [61] to the relevance of taking into account not only the individual perspective but also that of the group to understand the processes of therapeutic alliance. The results of this study are also in line with those found by [62], which pointed out that the therapeutic alliance as an individual within a group and the therapeutic alliance as a group may play different roles as predictors of therapeutic success. In our research, at the individual level, intraindividual processes were predictors of the participant’s alliance, but not of the group’s alliance. This implies that these intrapersonal processes explain the differences in alliance between participants, but not the alliance between groups. This reinforces the value of self-regulating processes for individual adaptation and could indicate that the key variables of group alliance are contextual and interpersonal factors. For example, authors such as [63,64], point out that certain group characteristics like cohesion, communication, and group size can affect the interaction between group members. We have also observed that emotion regulation and the difficulties of adaptation to divorce seem to affect the alliance of the therapist at the group level, reaffirming, therefore, the importance of these variables at both levels (individual and group) but with a differential character depending on who perceives the alliance (participant or therapist).

Finally, the present study also confirms, as already reported for other therapeutic modalities, that the participants’ symptomatology does not seem to be related to the therapeutic alliance [65,66]. In this case, the symptomatology was related to emotion regulation, but had no mediating effect on the establishment of the therapeutic alliance, either from the perspective of the participant or of the therapist. This result is maintained in the individual and group alliance.

In short, the results of this study have relevant clinical implications for therapists, insofar as they emphasize the need to take into account patients’ emotion regulation due to its relevant role in establishing the alliance therapeutic when there are severe interparental conflicts. Paying attention to patients’ emotion regulation in a therapeutic context could involve the therapist’s conscious effort to generate, through the therapeutic relationship, a corrective emotional experience that may contribute to the decline of the patient’s emotional arousal.

In post-divorce group interventions, it seems relevant to keep in mind that patients can initiate therapy with varying levels of emotion regulation, which also involves different therapeutic processes. This variability is a challenge for the therapist who, as noted, may feel overwhelmed. However, being aware of this will allow the therapist to direct efforts towards the acquisition of the patients’ emotion regulation strategies and favor the success of the interventions, as well as the reduction of therapeutic dropout.

Indirectly, the conscious approach of emotion regulation in therapy will have a fundamental impact on parenting [67], which could influence the children’s post-divorce adaptation.

We note several limitations in this study. The first is the small size of the sample. Although the sample is not negligible considering its specificity, a larger number of participants in the study would allow us to incorporate additional variables of the therapist or participants to the model without losing statistical robustness and would favor a greater generalization of the findings. A second limitation is the self-reported data. The SOFTA-o model allows an observational analysis [68] both of the therapist’s and the participant’s interventions, and its employment would help to understand the alliance in greater detail.

Future research could contemplate the incorporation of different moments throughout the process and after its completion, contributing to our understanding of the role of post-divorce emotion regulation in the results of the intervention. Also, the inclusion of contextual and interpersonal variables could further our understanding of group therapeutic alliance.

## Conclusions

In conclusion, the study underlines the important role of emotion regulation for the mental health and well-being of parents who are going through post-divorce difficulties with their ex-partners. This aspect should be considered when designing an adequate therapeutic alliance that favors the psychological adjustment not only of the parents but also of their children. This study makes an important contribution to the literature by providing preliminary evidence to suggest that parents’ emotion regulation and the psychological and emotional difficulties of adaptation to divorce can condition the therapeutic alliance, so it is necessary to consider these variables when designing post-divorce group intervention programs. In addition, the present study has highlighted the relevance of considering both the perspective of alliance of the therapist and of the participant and of addressing alliance from a group setting, which has proven to be a different context from the simple sum of its participants.
